# Evolutionary convergence of muscle architecture in relation to locomotor ecology in snakes

**DOI:** 10.1111/joa.13823

**Published:** 2023-02-02

**Authors:** Adrien Mathou, Xavier Bonnet, Karim Daoues, Rémi Ksas, Anthony Herrel

**Affiliations:** ^1^ Département Adaptations du Vivant, Bâtiment d'Anatomie Comparée UMR 7179 C.N.R.S/M.N.H.N. Paris France; ^2^ CEBC, UMR‐7372, CNRS‐Université de La Rochelle Villiers en Bois France; ^3^ La Ferme Tropicale Paris France; ^4^ Venomworld Saint‐Thibault‐des‐vignes France

**Keywords:** axial muscles, evolution, locomotion, muscle architecture, snake

## Abstract

The epaxial muscles in snakes are responsible for locomotion and as such can be expected to show adaptations in species living in different environments. Here, we tested whether the structural units that comprise the superficial epaxial muscles (semispinalis‐spinalis, SSP; longissimus dorsi, LD; iliocostalis, IC) were different in animals occupying similar habitats. To do so, we analyzed and compared the muscle architecture (mass, fiber length, and physiological cross‐sectional area) of the superficial epaxial muscle segments in snakes that differ in their habitat use (e.g., arboreal, terrestrial, and aquatic). Our results showed that arboreal species have on average longer muscles and tendons spanning more segments likely important during gap bridging. Moreover, aquatic snakes show relatively heavier semispinalis‐spinalis muscles with a greater cross‐sectional area. The longissimus dorsi muscles also showed a greater cross‐sectional area compared with terrestrial and especially arboreal snakes. Whereas the more strongly developed muscles in aquatic snakes are likely associated with the dense and viscous environment through which they move, the lighter muscles in arboreal snakes may provide an advantage when climbing. Future studies comparing other ecologies (e.g., burrowing snakes) and additional muscle units (e.g., multifidus; hypaxial muscles) are needed to better understand the structural features driving variation in locomotor performance and efficiency in snakes.

## Graphical abstract

Snakes with different ecologies differ in the axial musculature with aquatic species having more robust muscles. Arboreal species on the other hand typically have longer muscle‐tendon units.

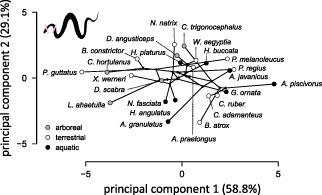

## 1 INTRODUCTION

Despite the absence of limbs (Da Silva et al., 2018; Jayne, 2020) snakes are an extremely successful group of squamates with nearly 4000 described species (Uetz, 2022). Not only are snakes a species‐rich group, they also occupy a wide variety of environments ranging from truly marine species, over terrestrial, arboreal, and specialized fossorial taxa. The occupation of these different ecological niches has gone hand in hand with morphological and functional adaptations to the constraints imposed by these environments. For example, aquatic snakes show striking convergent specializations in head and tail size and shape in relation to the dense and viscous medium in which they move and feed (Aubret & Shine, 2008; Brischoux & Shine, 2011; Segall et al., 2016). Similarly, arboreal, aquatic, and terrestrial snakes differ in the position of the heart along the body in response to the need to pump blood against gravity in climbing arboreal species, for example (Gartner et al., 2010).

In snakes, locomotion consists of four major types that are used depending on the constraints of the medium: rectilinear, lateral undulation, sidewinding, and concertina (Jayne, 2020). Whereas lateral undulation is used in most environments, concertina, rectilinear locomotion, and sidewinding are more specialized locomotor modes used in specific contexts (Jayne, 2020). Previous studies have suggested that the most superficial epaxial muscles, namely semispinalis‐spinalis (SSP), longissimus dorsi (LD), and iliocostalis (IC) muscle groups are the main drivers of locomotion (Gans, 1986; Gasc, 1981; Gasc et al., 1989; Jayne, 1988a, 1988b; Moon & Gans, 1998; Mosauer, 1932). These muscles represent roughly 65% of the axial muscle mass (Ruben, 1977) and their cross‐section scales isometrically with overall body mass (Moon & Candy, 1997). Yet, variation in the mass and length of these muscles has been documented and has been suggested to be related to the locomotor environment and predation mode (Herrel et al., 2011; Jayne, 1982; Lourdais et al., 2005; Moon, 2000; Penning, 2018; Penning & Moon, 2017; Ruben, 1977; Young, 2010).

However, only a few studies have quantitatively explored differences in the muscle architecture of the epaxial musculature (Jayne, 1982; Penning, 2018) and even fewer have done so in a comparative phylogenetic framework. Here, we test whether the structural units that make up the superficial epaxial muscles (SSP, LD, IC) have evolved convergently in species subjected to similar locomotor constraints. We analyzed and compared the muscle architecture (mass, fiber length, and physiological cross‐sectional area) of the muscle segments of the superficial epaxial muscles (SSP, LD, IC) in snakes that differ in their habitat use (i.e., arboreal, terrestrial, and aquatic). We specifically focus on aquatic species given that (1) a significant number of species from different families have convergently evolved an aquatic or semi‐aquatic lifestyle (Murphy, 2007; Pauwels et al., 2008) and (2) the strong physical constraints (drag) associated with the aquatic medium (i.e., viscosity and density) that will impact the kinematics and energetics of locomotion (Vogel, 1994). We predict that aquatic species should have stronger muscles to move the body against a dense fluid. Conversely, we predict arboreal species to have longer and lighter muscles with longer tendons allowing them to bridge gaps in the complex three‐dimensional environment they live in (Jayne, 1982; Jayne & Riley, 2007; Lillywhite et al., 2000).

## 2 MATERIAL AND METHODS

### 2.1 Species

We characterized the musculature of 24 species of snakes belonging to nine families: *Colubridae, Viperidae, Natricidae, Acrochordidae, Dipsadidae, Boidae, Pythonidae, Homalopsidae, and Elapidae* (Table 1). We examined 1 individual per species except for *Pantherophis guttatus* (*N* = 2), *Homalopsis buccata* (*N* = 2), and *Python regius* (*N* = 2). Species were selected to encompass a diversity of life‐styles across the different families. We were specifically interested in adaptations to an aquatic life‐style and focused our sampling on this ecology. We classified semi‐aquatic species as aquatic in our analyses as we had too few semi‐aquatic species to treat them as a separate group.

**TABLE 1:** Species used, including the number of individuals dissected, the ecological classification used, the family, snout‐vent length (SVL), body mass, and mid‐body diameter.

Species	*N*	Ecology	Family	SVL (cm)	Mass (g)	Diameter (mm)
*Acanthophis praelongus*	1	T	Elapidae	57.2	192	27
*Acrochordus granulatus*	1	AQ	Acrochordidae	48.1	77	15.91
*Acrochordus javanicus*	1	AQ	Acrochordidae	68	286	27.1
*Agkistrodon piscivorus*	1	AQ	Viperidae	91	1046	41.5
*Boa constrictor*	1	T	Boidae	39.5	45	17.2
*Bothrops atrox*	1	T	Viperidae	61.3	297	28.95
*Corallus hortulanus*	1	AR	Boidae	67.1	27	9
*Craspedocephalus trigonocephalus*	1	AR	Viperidae	79.7	240	22
*Crotalus adamanteus*	1	T	Viperidae	92.3	578	33
*Crotalus ruber*	1	T	Viperidae	113.3	677	25
*Dasypeltis scabra*	1	AR	Colubridae	73.7	74	15.91
*Dendroaspis angusticeps*	1	AR	Elapidae	140	395	21
*Grayia ornata*	1	AQ	Colubridae	105.3	538	23.01
*Helicops angulatus*	1	AQ	Dipsadidae	35.7	46	11.98
*Homalopsis buccata*	2	AQ	Homalopsidae	67.2 ± 24.1	147 ± 13	20.69 ± 0.32
*Hydrophis platurus*	1	AQ	Elapidae	65.2	125	20.7
*Leptophis ahaetulla*	1	AR	Colubridae	51.5	13	5.02
*Natrix natrix*	1	T	Natricidae	75.1	233	21.18
*Nerodia fasciata*	1	AQ	Natricidae	47.5	50	15.6
*Pantherophis guttatus*	2	T	Colubridae	48.05 ± 1.7	19 ± 18	11.94 ± 1.59
*Pituophis melanoleucus*	1	T	Colubridae	114.1	391	24.93
*Python regius*	2	T	Pythonidae	95.5	647	45
*Walterinnesia aegyptia*	1	T	Elapidae	87	222	17
*Xenodon werneri*	1	T	Dipsadidae	46.8	51	14.32

Abbreviations: AQ, aquatic; AR, arboreal; T, terrestrial.

### 2.2 Dissection

All individuals were weighed (± 1 g; Kern PNJ). We measured snout‐vent length and tail length with a string and a ruler to the nearest mm. Body diameter at mid body was measured using manual Vernier calipers (Hilka Tools 150 mm, ± 0.02 mm). Three sections of equal size (from 1 to 10 cm depending on the size of the snake) were analyzed given that snakes are known to exhibit longitudinal variation in the anatomy of the epaxial muscles (Pregill & Pregill, 1977). We dissected the epaxial muscles at (1) the anterior body at about one fourth of the total length of the snake, (2) midbody, and (3) the posterior body just anterior to the cloaca.

**FIGURE 1:**
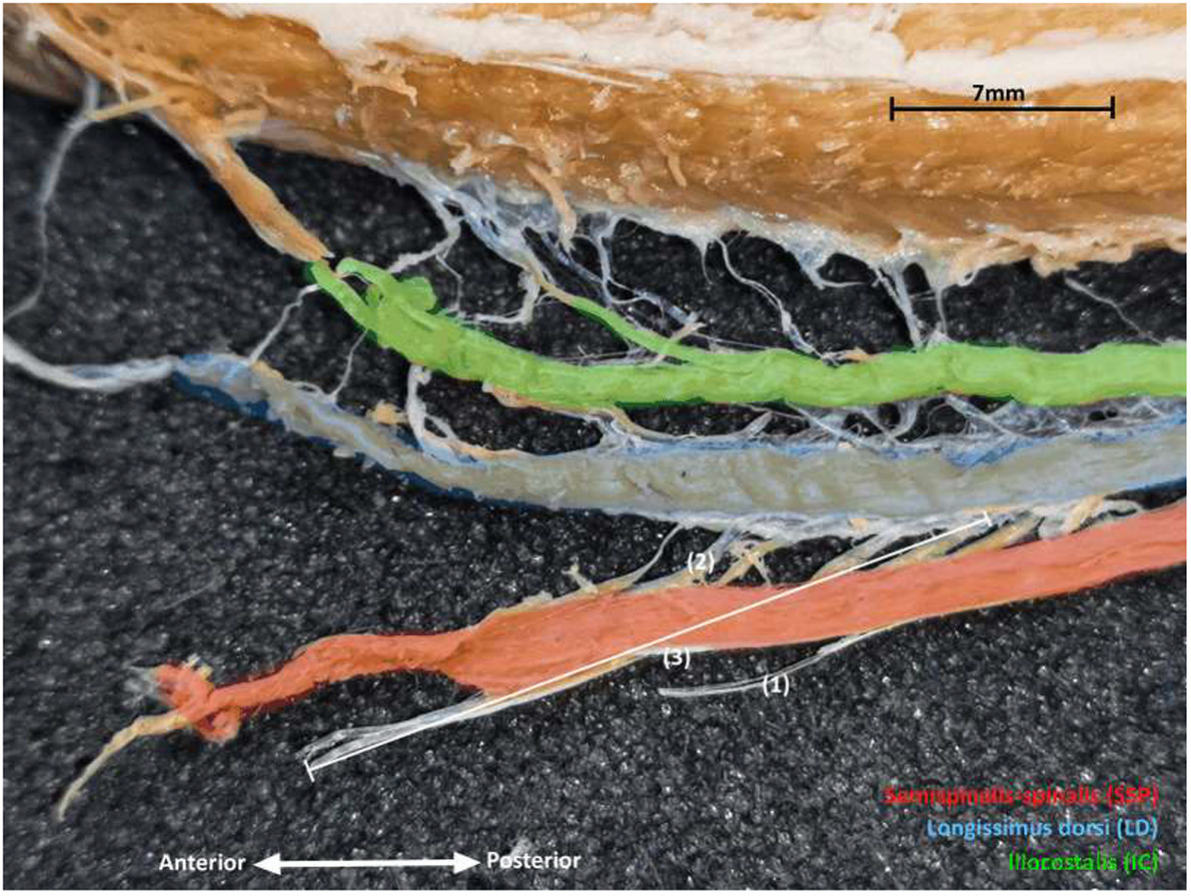
Photograph of the axial muscles in *Pantherophis guttatus*. 1, upper tendon of the semispinalis‐spinalis; 2, lower tendon of the semispinalis‐spinalis; and 3, semispinalis‐spinalis muscle‐tendon unit. The semispinalis‐spinalis is visible at the bottom, the longissimus in the middle and the iliocostalis at the top of the picture. Anterior is to the right.

We measured the longitudinal extent (number of vertebrae, including the vertebrae of origin and insertion for muscles or tendons inserting directly on the vertebrae) and the size (in cm, with a ruler) of the upper (i.e., anterior tendon of the semispinalis‐spinalis; posterior tendon of the iliocostalis) tendons, the muscle bellies, and the lower (posterior tendon of the semispinalis‐spinalis; anterior tendon of the iliocostalis) tendons of the semispinalis‐spinalis, the longissimus dorsi, and the iliocostalis muscles (Figure 1). Note that the posterior tendons of the semispinalis‐spinalis and the iliocostalis are attached to the longissimus dorsi. By consequence, the posterior (lower) tendon of the semispinalis‐spinalis is also the upper tendon of the longissimus and the posterior (upper) tendon of the iliocostalis is also the lower tendon of the longissimus dorsi.

Next, muscles were removed and weighed on a precision balance (Mettler AE100, precision ± 0.0001 g). We then measured the insertion angle of the fibers on the tendons by using a binocular scope (Leica Wild M3Z) with camera lucida (Motic MLC‐150C) and a protractor to obtain the pennation angle. Next, we submerged the muscles in a 30% aqueous nitric acid solution for 24–48 h. After digestion of the connective tissue, fibers were teased apart and the nitric was replaced by a 50% aqueous glycerol solution to stop further digestion. The fibers were then viewed, redrawn under a binocular scope (Leica Wild M3Z) with camera lucida (Motic MLC‐150C), photographed and measured in ImageJ (Abramoff et al., 2004). Muscle cross‐sectional area was measured by first calculating muscle volume from muscle mass using a muscle density of 1.06 gcm^−3^ (Mendez & Keys, 1960) and then dividing this by fiber length while correcting for pennation angle by multiplying this by the cosine of the pennation angle.

### 2.3 Statistical analyses

**FIGURE 2:**
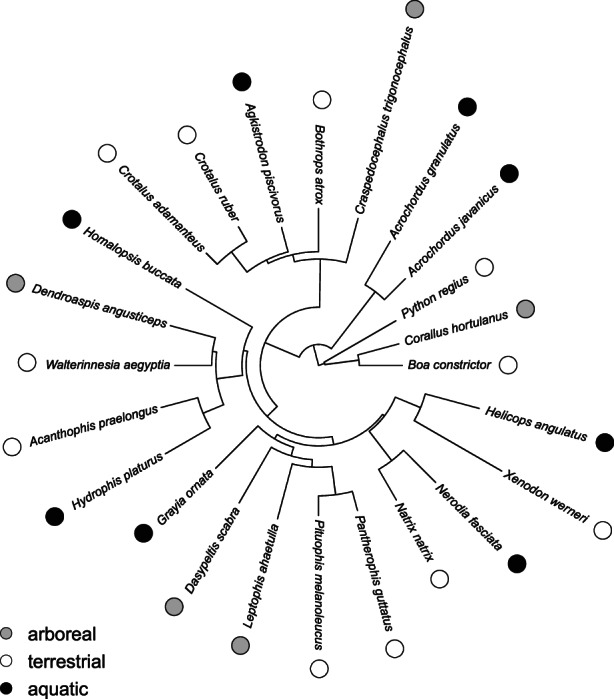
Phylogenetic relationships between the species included in this study (based on Pyron et al., 2013). Ecological groups are indicated by colored circles next to the species' names.

All data were Log_10_‐transformed before analysis. First, we performed a descriptive analysis on the segment lengths to explore variation in the extent of the muscle‐tendon units by exploring variation in the number of vertebrae spanned by each muscle and tendon. Next, we ran four separate principal component analysis (PCA) in R (packages FactomineR, FactoExtra) on the muscle architecture data. One for the overall data including snout‐vent length, body mass, mid‐body diameter, and the overall mass and length of the three muscles. Next, we ran separate PCAs in each of the three body regions (anterior, mid‐body, and posterior) including absolute values of the mass, fiber length, and physiological cross‐sectional area of each muscle segment. We cropped the phylogeny provided by Pyron et al. (2013) to include only the species included in our data set (Figure 2) and used it to create a phylomorphospace (Revell & Collar, 2009) in Phytools (Revell, 2012). We next calculated the phylogenetic signal in our data. To do so we calculated a multivariate Blomberg's K (Kmult) in the package Geomorph (Adams & Otárola‐Castillo, 2013).

We subsequently performed a MANCOVA (multivariate analysis of covariance) on the muscle architecture values (fiber length, muscle length, muscle mass, PCSA) for the three body parts (anterior, mid‐body, posterior) separately to test whether species with different locomotor ecologies differed in their muscle architecture. Given that significant differences were observed, we then ran univariate analyses of variance to test which variables differed between groups. Next, we calculated unstandardized residuals of a regression of each muscle variable on snout‐vent length and used them as input for ANOVAs coupled to Tukey posy‐hoc tests. For variables showing significant differences, we inspected the mean residuals by ecological group. Finally, we performed a phylogenetic MANCOVAs in mvMORPH (Clavel et al., 2015) and phylogenetic ANCOVAs using phytools (Revell, 2012) to test whether our results held when taking into account the phylogenetic relationships. To explore which groups differed from one another we ran PGLS regressions using the package Caper in R (Orme et al., 2013) and used the residuals (independent of variation in overall size) as input for ANOVAs. When significant differences between groups were detected, we inspected the means of the residuals per ecological group to assess the directionality of these differences.

All analyses were performed in R (R Core Team, 2020) and *α* was set at 0.05.

## 3 RESULTS

Quantitative data on muscle segment lengths and muscle architecture for each individual are presented in Tables S1 and S2.

### 3.1 Variation in muscle origin and insertion

**FIGURE 3:**
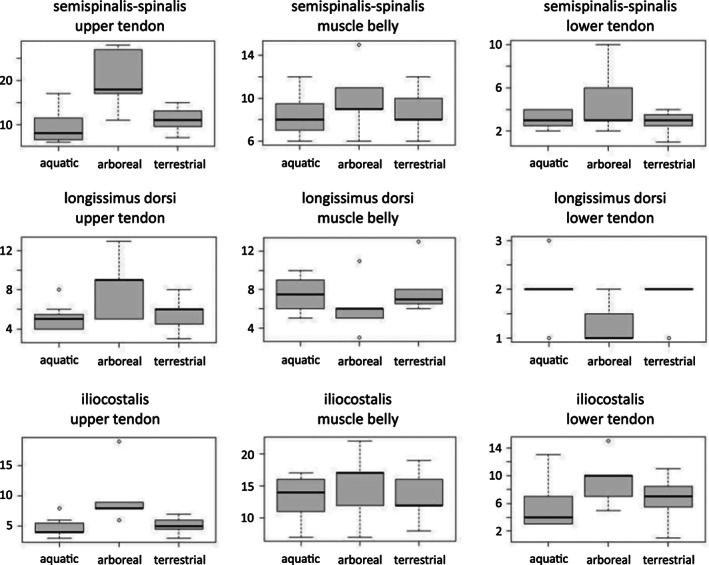
Box plots illustrating the longitudinal extent of the tendons and contractile part of the axial muscles for the different ecological groups. Values are the number of vertebrae crossed.

In arboreal species, the lower and upper tendons as well as the contractile part of the semispinalis‐spinalis and iliocostalis muscles generally span more vertebrae (Table S1, Figure 3, Figures S1–S3). However, the contractile part of the longissimus dorsi and its lower tendon extends over more vertebrae in terrestrial and aquatic species. The upper tendons of the longissimus dorsi, however, do extend further in arboreal species (Figures S1–S3). Significant amounts of variation are present within each ecological group (Figure 3, Figures S1–S3).

### 3.2 Variation in muscle architecture

The principal component analysis performed on the overall data set including all body parts (Table S2) extracted two axes jointly explaining over 95% of the variation in the data set. The first component is a descriptor of overall size with larger species with heavy muscles having positive loadings (Table S3). The second axis differentiates between species that are overall longer versus more robust (Table S3).

**FIGURE 4:**
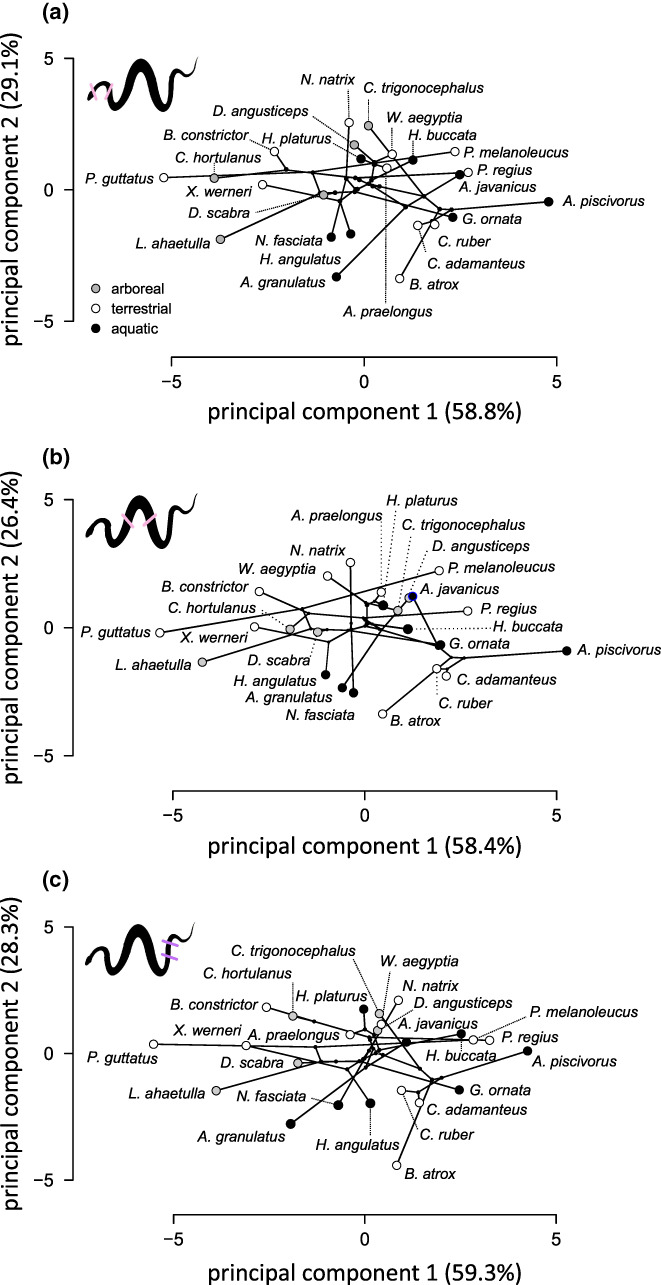
Results of the principal component analysis performed for the anterior (a), mid‐body (b) and posterior part of the body (c). The phylogeny is plotted in the morphospace. Colored symbols illustrate different ecologies. Black, aquatic; white, terrestrial; grey, arboreal.

The PCAs performed on the muscle architecture data for the three body parts separately show similar results (Figure 4). Overall, the first two axes explain between 84.4% and 87.9% of the variation in the data set (Table 2). The first axis differentiates species with heavy epaxial muscles with a greater cross‐sectional area such as *Agkistrodon piscivorus* or *P. regius* that cluster to the right of the plot from more slender species that have a less developed (i.e., lower mass and cross‐sectional area) musculature like *P. guttatus* or *Leptophis ahaetulla* to the left. The second axis is mostly determined by variation in muscle fiber length with species with longer fibers such as *Natrix natrix* clustering towards the positive side of the axis.

**TABLE 2:** Loading of the original variables on the different principal components. A separate PCA was performed for each body region.

	PC1 (58.8%)	PC2 (29.1%)
*Anterior body*		
SSP segment mass (g)	**0.95**	0
LD segment mass (g)	**0.95**	0.07
IC segment mass (g)	**0.92**	0.19
Mean length SSP fibers (cm)	0.25	**0.80**
Mean length IC fibers (cm)	0.29	**0.89**
Mean length LD fibers (cm)	0.21	**0.88**
PCSA SSP (cm^2^)	**0.89**	−0.36
PCSA LD (cm^2^)	**0.91**	−0.34
PCSA IC (cm^2^)	**0.89**	−0.30

	PC1 (58.4%)	PC2 (26.4%)
*Mid‐body*		
SSP segment mass (g)	**0.93**	0.02
LD segment mass (g)	**0.94**	0.09
IC segment mass (g)	**0.96**	0.03
Mean length SSP fibers (cm)	0.34	**0.72**
Mean length IC fibers (cm)	0.43	**0.83**
Mean length LD fibers (cm)	0.28	**0.80**
PCSA SSP (cm^2^)	**0.82**	−0.37
PCSA LD (cm^2^)	**0.90**	−0.28
PCSA IC (cm^2^)	**0.81**	−0.52

	PC1 (59.3%)	PC2 (28.3%)
*Posterior body*		
SSP segment mass (g)	**0.95**	−0.08
LD segment mass (g)	**0.96**	0.12
IC segment mass (g)	**0.94**	0.03
Mean length SSP fibers (cm)	0.45	**0.70**
Mean length IC fibers (cm)	0.41	**0.84**
Mean length LD fibers (cm)	0.24	**0.91**
PCSA SSP (cm^2^)	**0.80**	−0.45
PCSA LD (cm^2^)	**0.94**	−0.23
PCSA IC (cm^2^)	**0.79**	−0.47

*Note*: Variables loading highly (>0.7) are indicated in bold.

Abbreviations: IC, iliocostalis; LD, latissimus dorsi; PCSA, physiological cross‐sectional area; SSP, semispinalis‐spinalis.

### 3.3 Ecological differences

Traditional MANCOVAs showed a significant effect of snout‐vent length on muscle architecture for all three body regions (Table S4). Ecological groups differed in the architecture of the muscles at the level of the mid‐body (Pillai = 1.25; *F*_18,26_ = 2.42; *p* = 0.019). Differences between ecological groups were, however, not significant for the anterior (Pillai = 1.00; *F*_18,26_ = 1.44; *p* = 0.19) and posterior (Pillai = 1.01; *F*_18,26_ = 1.48; *p* = 0.18) body regions (Table S4). Subsequent univariate ANCOVAs performed for the mid‐body segment showed that the fiber lengths of the semispinalis‐spinalis (*F*_2,20_ = 4.29; *p* = 0.028), the PCSA of the semispinalis‐spinalis (*F*_2,20_ = 6.39; *p* = 0.007) and the mass of the longissimus dorsi (*F*_2,20_ = 4.15; *p* = 0.031) were different between ecological groups (Table S5). Post‐hoc tests showed that the PCSA of the semispinalis‐spinalis muscle at mid‐body was different with aquatic snakes having a greater cross‐sectional area. Moreover, the mass of the longissimus dorsi differed between arboreal and aquatic snakes with aquatic snakes having heavier muscles (Tables S6 and S7).

The phylogenetic MANCOVAs showed a significant impact of body size on muscle architecture (Table 3). Moreover, differences were significant between ecological groups for all segments (anterior body: Wilks' = 0.09, *F*_18,20_ = 2.53, *p* = 0.023; mid‐body: Wilks' = 0.06, *F*_18,20_ = 3.28, *p* = 0.0059; posterior body: Wilks' = 0.07, *F*_18,20_ = 2.92, *p* = 0.011). Interactions between snout‐vent length and muscle architecture were significant for the anterior and posterior body regions suggesting that the relationships between size (SVL) and muscle architecture differ between groups (Table 3). Subsequent univariate phylogenetic ANCOVAs showed that for the anterior part of the body differences were significant for the mass of the semispinalis‐spinalis and the longissimus dorsi as well as for the PCSA of the longissimus dorsi muscle (Table 4). At mid‐body, differences were significant for the mass of the semispinalis‐spinalis and longissimus only. For the posterior body, the mass of the longissimus dorsi and the PCSA of the longissimus dorsi were the only variables that differed between ecological groups (Table 4). Post‐hoc analysis on the residual data derived from the PGLS regressions showed differences between all ecological groups in the mass of the semi‐spinalis‐spinalis and longissimus dorsi in the anterior part of the body. Moreover, the PCSA of the longissimus dorsi was different between aquatic species on the one hand and arboreal and terrestrial species on the other hand (Table S8). At mid body, aquatic snakes were again different from other ecological groups in the mass of the semispinalis‐spinalis. However, for the longissimus at mid‐body aquatic snakes differed only from that in arboreal species (Table S8). Finally, at the posterior body, aquatic snakes differed again from the two other groups in both the mass and the PCSA of the longissimus dorsi. Overall, aquatic species had heavier muscles with a greater force generating capacity.

**TABLE 3:** Results of the phylogenetic MANCOVAs for the different body regions.

	Wilks'	Approx. *F*	Num *df*	Den *df*	*p*
Anterior body					
**SVL**	0.12	8.13	9	10	0.0015*
**Ecology**	0.093	2.54	18	20	0.023*
**Interaction**	0.093	2.54	18	20	0.023*
Mid‐body					
**SVL**	0.26	3.22	9	10	0.042*
**Ecology**	0.064	3.29	18	20	0.0059*
Interaction	0.36	0.73	18	20	0.74
Posterior body					
**SVL**	0.13	7.75	9	10	0.0018*
**Ecology**	0.076	2.92	18	20	0.011*
**Interaction**	0.091	2.57	18	20	0.022*

*Note*: Bold variables and asterisks indicate significant differences.

**TABLE 4:** Results of the univariate phylogenetic ANCOVAs for the different body regions testing for differences between ecological groups.

Dependent variable	*df*	Sum of square	Mean sum of square	*F*	*p*
*Anterior body*					
**SSP mass (g)**	6	0.400	0.022	4.178	0.014*
**LD mass (g)**	6	0.475	0.026	3.809	0.020*
IC mass (g)	6	0.640	0.035	1.753	0.182
SSP fiber length (cm)	6	0.735	0.040	0.546	0.703
LD fiber length (cm)	6	1.124	0.062	0.366	0.829
IC fiber length (cm)	6	1.042	0.057	0.337	0.849
SSP PCSA (cm^2^)	6	0.791	0.044	2.090	0.124
**LD PCSA (cm** ^**2**^ **)**	6	0.617	0.034	3.117	0.041*
IC PCSA (cm^2^)	6	0.738	0.041	2.136	0.118
*Mid‐body*					
**SSP mass (g)**	6	0.359	0.020	3.830	0.020*
**LD mass (g)**	6	0.418	0.023	3.102	0.041*
IC mass (g)	6	0.630	0.035	1.644	0.206
SSP fiber length (cm)	6	0.738	0.041	0.177	0.947
LD fiber length (cm)	6	0.958	0.053	0.731	0.582
IC fiber length (cm)	6	0.804	0.044	0.739	0.577
SSP PCSA (cm^2^)	6	0.827	0.046	2.373	0.090
LD PCSA (cm^2^)	6	0.563	0.031	2.264	0.102
IC PCSA (cm^2^)	6	1.017	0.056	1.086	0.392
*Posterior body*					
SSP mass (g)	6	0.478	0.026	2.601	0.070
**LD mass (g)**	6	0.501	0.027	3.170	0.038*
IC mass (g)	6	0.491	0.027	1.882	0.157
SSP fiber length (cm)	6	0.642	0.035	1.036	0.415
LD fiber length (cm)	6	1.083	0.060	0.352	0.838
IC fiber length (cm)	6	1.027	0.057	0.438	0.779
SSP PCSA (cm^2^)	6	0.994	0.055	0.873	0.498
**LD PCSA (cm** ^**2**^ **)**	6	0.562	0.031	3.329	0.033*
IC PCSA (cm^2^)	6	0.662	0.036	1.822	0.168

*Note*: Bolded variables and asterisks indicate significant differences.

Abbreviations: IC, iliocostalis; LD, latissimus dorsi; PCSA, physiological cross‐sectional area; SSP, semispinalis‐spinalis.

### 3.4 Phylogenetic signal

Blomberg's multivariate *K* showed moderate but non‐significant phylogenetic signal in the dataset for each of the body regions analyzed (anterior: *K* = 0.40, *p* = 0.78; mid‐body: *K* = 0.42, *p* = 0.71; posterior: *K* = 0.371, *p* = 0.87). This is further illustrated in the phylomorphospace where phylogenetically distant species of the same ecology tend to group together. For example, the two Dipsadidae in our analysis, *Xenodon werneri* and *Helicops angulatus* fall in different parts of the morphospace close to species with a similar ecology (Figure 4).

## 4 DISCUSSION

In our data set, the phylogenetic signal in the muscle architecture data was not significant. This is quite unexpected as axial muscle anatomy has been previously used as a systematic character in snakes (Mosauer, 1935). Mosauer (1935) recognized three distinct types characterizing boids, colubrids, and vipers with little variation within each group despite the fact that he had examined species with very different ecologies. However, later authors (Auffenberg, 1966; Gasc, 1981) have suggested this to be oversimplified and the studies of Ruben (1977) and Jayne (1982) clearly demonstrated variation in the semi‐spinalis‐spinalis system in relation to ecology. Our results similarly suggest that habitat use, and more specifically locomotion in different habitats, is an important driver of muscle anatomy in snakes. These results echo findings for other taxa. For example, Omura (2019) demonstrated that the abdominal muscles were better developed in terrestrial salamanders as they function to retain the visceral mass against gravity. Conversely, in aquatic salamanders, the lateral hypaxial muscles that contribute to body flexion are more strongly developed (Omura, 2019; Omura et al., 2014, 2015).

In our data set, aquatic snakes stand out from terrestrial and especially arboreal snakes in having generally shorter, heavier, and more forceful epaxial muscles, particularly the longissimus dorsi. These results are in line with previous results for snakes showing that the musculoskeletal system of aquatic snakes is quite different from terrestrial and arboreal ones. For example, the costocutaneus muscles that help flatten the body during swimming have been shown to have a more dorsal insertion in fully aquatic snakes (Voris & Jayne, 1976). Moreover, seminal work by Jayne (1982) showed that arboreal snakes had longer tendons in the semispinalis‐spinalis compared with aquatic snakes, similar to our observations. The elongation of the tendons of the epaxial muscles likely brings a mechanical advantage by increasing the muscle lever arms and by consequence the cantilever ability of the body (Jayne, 1986), which may provide an advantage during gap bridging (Jayne & Riley, 2007). Our data also show that the muscle belly of the iliocostalis muscle is also longer in arboreal snakes suggesting that arboreal species may be characterized by an overall elongation and reduction in weight of the axial muscle system. In contrast, aquatic species appear to have shorter tendons and muscles and more robust and heavier muscles.

Unexpectedly, our quantitative analyses detected no differences in iliocostalis muscle architecture between ecological groups, suggesting that this muscle is the least variable of the axial muscles. The semispinalis‐spinalis and longissimus dorsi systems, on the other hand, appear to be much more strongly influenced by ecology. This influence may be explained in part by the fact that semispinalis‐spinalis plays a different role in locomotion and other movements (Auffenberg, 1966; Gasc et al., 1989; Jayne, 1982; Moon & Gans, 1998; Pregill & Pregill, 1977; Ruben, 1977). For example, the semispinalis and longissimus muscles are also important for striking in snakes as they are the two main extensors of the spine (Young, 2010). These muscles may therefore be involved in different functions and as such may be subject to trade‐offs. Moreover, it has been suggested that the semispinalis‐spinalis and iliocostalis muscles may function to modulate the activity of the more posterior longissimus and as such may show different constraints (Moon & Gans, 1998). Previous studies have shown that muscle activity propagates from the front to the back of the body and that the longissimus dorsi is activated later than other muscles (Gasc et al., 1989; Jayne, 1988a, 1988b; Moon & Gans, 1998). This suggests functional differences between the different axial muscle groups.

Interestingly, not all body parts showed the same signal with the posterior part showing differences only in the longissimus dorsi. Auffenberg (1958) discussed columnar variation and showed that the anterior muscle segments are generally shorter but did not discuss differences between species with different locomotor ecologies. Unfortunately, few authors have discussed the variation in muscle architecture along the vertebral column in detail (but see Nicodemo, 2012; Pregill & Pregill, 1977). The different signal observed in our data suggests different functional roles for different body parts and suggest that mainly the anterior and mid‐body axial muscles may be involved in generating the power for locomotion. The posterior body may be involved in grasping (arboreal species), controlling the amplitude of the rear of the body during undulations, or in other functions beyond locomotion (copulation, housing of the reproductive organs) that may constrain the development of the axial muscles.

Overall, our results show that aquatic species have shorter and heavier muscle segments than arboreal or terrestrial species. This makes intuitive sense as they have to move through a denser and more viscous environment (Vogel, 1994). Moreover, there is a fundamental difference between muscle activity during aquatic and terrestrial lateral undulation (Jayne, 1985, 1988a, 1988b). Whereas during both swimming and terrestrial locomotion all muscles (SSP, IC, and LD) are activated simultaneously, the wave of activation propagates faster down the body than the kinematic wave during swimming (Jayne, 1985). This suggests that the functional demands on the axial muscles may differ for species that spend a greater proportion of their time swimming.

Although our results suggest a strong relationship between axial muscle anatomy and the locomotor environment in snakes, it would be important to enlarge the data set. Both adding additional species as well as species with different ecologies including fossorial species would be important. Similarly, exploring whether a similar signal is present in other muscles or not would be important. Muscles like the multifidus, hypaxial muscles, or levatores costae are known to play an important role during locomotion and need to be explored. Finally, our study considers only a few individuals per species and thus neglects potential intraspecific variability and sexual dimorphism (Bonnet et al., 1998; Penning, 2018). Exploring how individuals within a population, or how populations that differ in their ecology, vary could help better understand the selective pressures driving the observed variation in anatomy.

## AUTHOR CONTRIBUTIONS

Adrien Mathou and Anthony Herrel conceived the study. Adrien Mathou performed data acquisition and analysis. Xavier Bonnet, Karim Daoues, Rémi Ksas, and Anthony Herrel provided resources. Adrien Mathou drafted the manuscript with the help of Anthony Herrel. All authors read and provided critical feedback on the first draft of the manuscript.

## Supporting information


Appendix S1

## Data Availability

All data are available in the paper and in the supplementary information.
