# Supplementary material for: Evolutionary convergence of muscle architecture in relation to locomotor ecology in snakes
Source: J Anat. 2023 Feb 2;242(5):862–71. doi: 10.1111/joa.13823 (PMC10093152; doi:10.1111/joa.13823)
Supplement: Supplementary file 1 — Appendix S1 [file JOA-242-862-s001.zip › JOA_13823_Figure S1 - Aquatic.pdf]

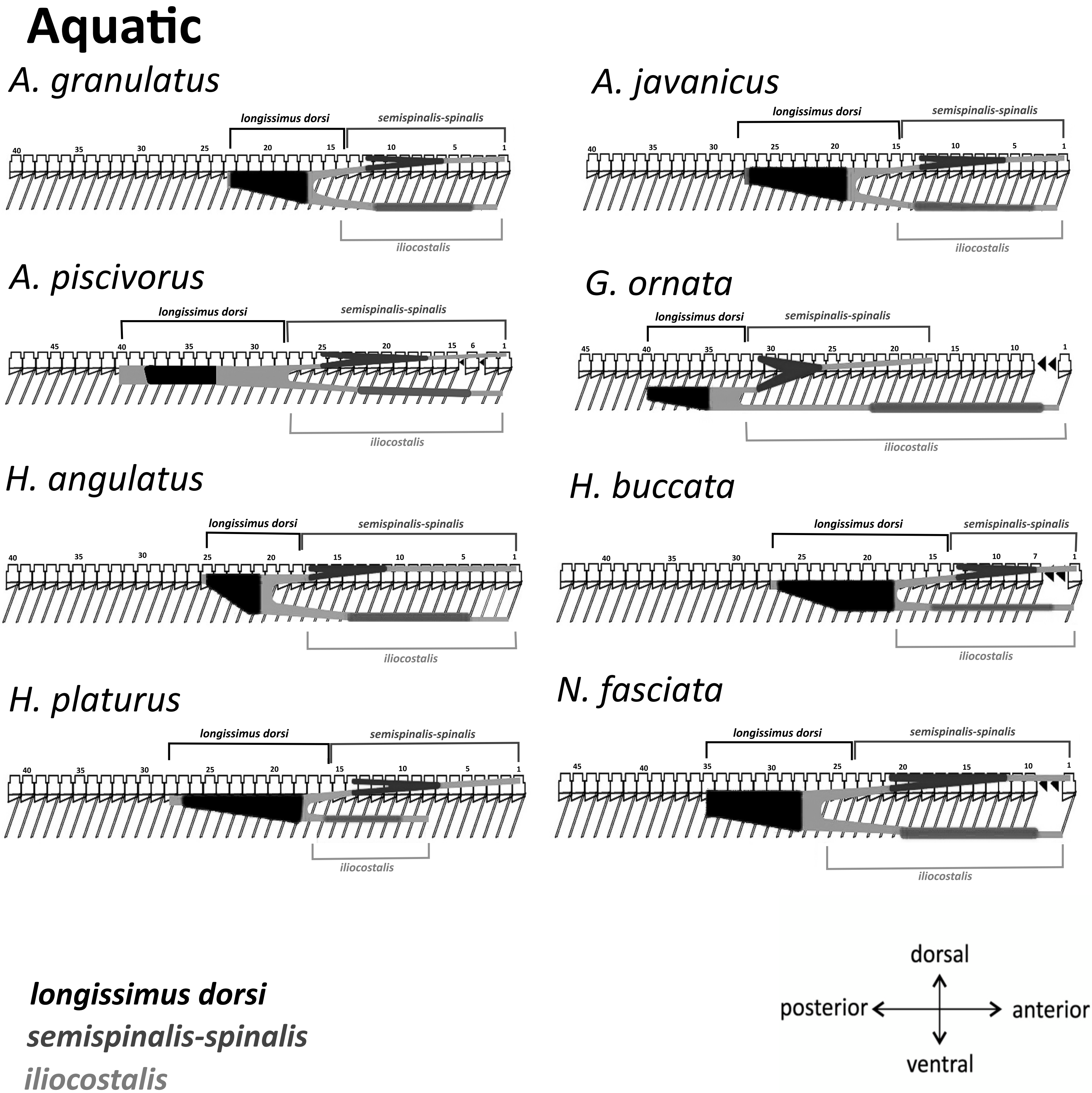
Aquatic
A. granulatus
longissimus dorsi
semispinalis-spinalis
iliocostalis
A. javanicus
longissimus dorsi
semispinalis-spinalis
iliocostalis
A. piscivorus
longissimus dorsi
semispinalis-spinalis
iliocostalis
G. ornata
longissimus dorsi
semispinalis-spinalis
iliocostalis
H. angulatus
longissimus dorsi
semispinalis-spinalis
iliocostalis
H. buccata
longissimus dorsi
semispinalis-spinalis
iliocostalis
H. platurus
longissimus dorsi
semispinalis-spinalis
iliocostalis
N. fasciata
longissimus dorsi
semispinalis-spinalis
iliocostalis
longissimus dorsi
semispinalis-spinalis
iliocostalis
dorsal
posterior
anterior
ventral
