# Supplementary material for: Evolutionary convergence of muscle architecture in relation to locomotor ecology in snakes
Source: J Anat. 2023 Feb 2;242(5):862–71. doi: 10.1111/joa.13823 (PMC10093152; doi:10.1111/joa.13823)
Supplement: Supplementary file 1 — Appendix S1 [file JOA-242-862-s001.zip › JOA_13823_Figure S2 - Arboreal.pdf]

Arboreal

C. hortulanus

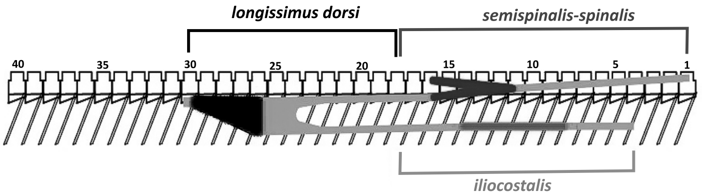

C. trigonocephalus

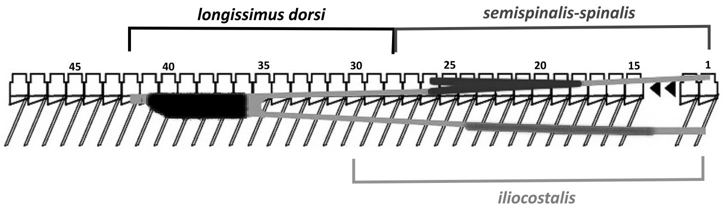

D. angusticeps

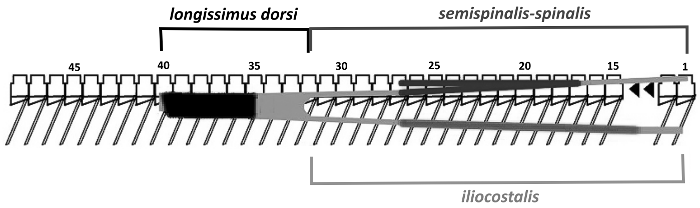

D. scabra

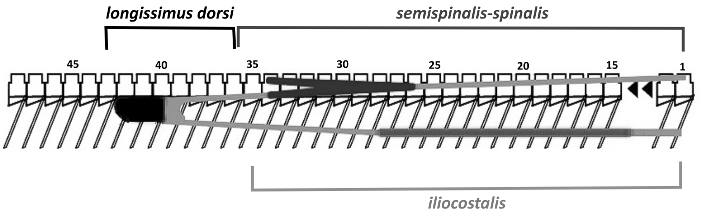

L. ahaetulla

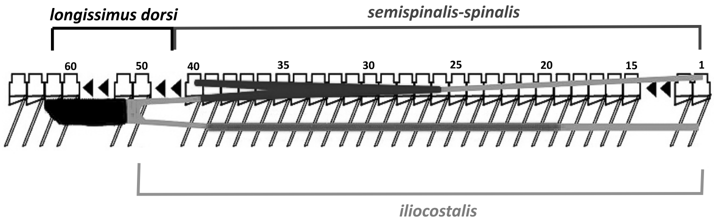

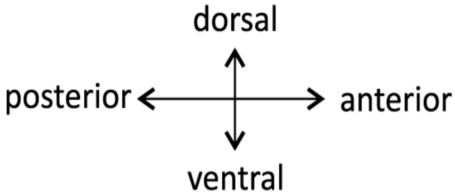

longissimus dorsi

semispinalis-spinalis

iliocostalis
