# Supplementary material for: Evolutionary convergence of muscle architecture in relation to locomotor ecology in snakes
Source: J Anat. 2023 Feb 2;242(5):862–71. doi: 10.1111/joa.13823 (PMC10093152; doi:10.1111/joa.13823)
Supplement: Supplementary file 1 — Appendix S1 [file JOA-242-862-s001.zip › JOA_13823_Figure S3 - Terrestrial.pdf]

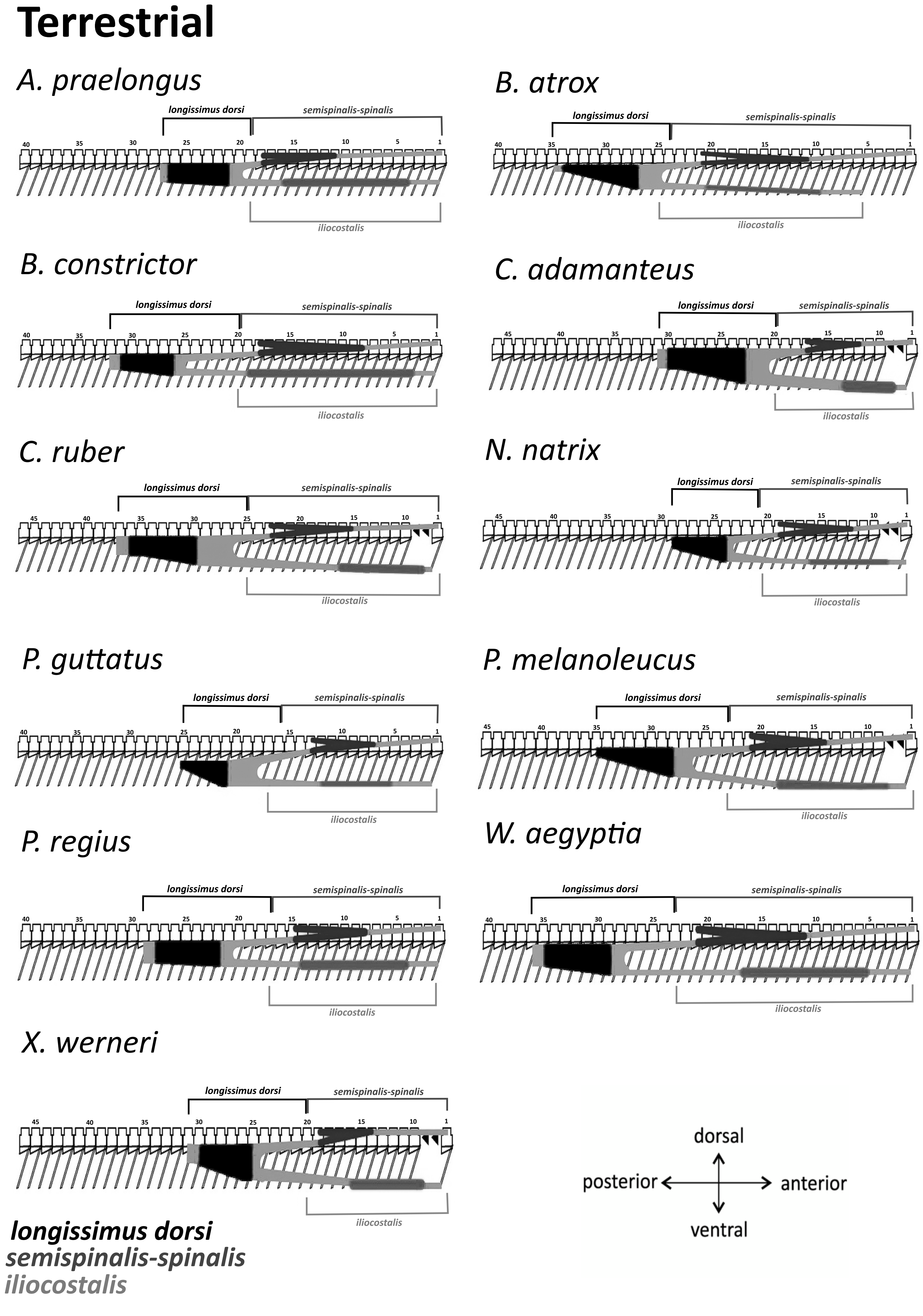

Terrestrial
A. praelongus
longissimus dorsi
semispinalis-spinalis
iliocostalis
B. atrox
longissimus dorsi
semispinalis-spinalis
iliocostalis
B. constrictor
longissimus dorsi
semispinalis-spinalis
iliocostalis
C. adamanteus
longissimus dorsi
semispinalis-spinalis
iliocostalis
C. ruber
longissimus dorsi
semispinalis-spinalis
iliocostalis
N. natrix
longissimus dorsi
semispinalis-spinalis
iliocostalis
P. guttatus
longissimus dorsi
semispinalis-spinalis
iliocostalis
P. melanoleucus
longissimus dorsi
semispinalis-spinalis
iliocostalis
P. regius
longissimus dorsi
semispinalis-spinalis
iliocostalis
W. aegyptia
longissimus dorsi
semispinalis-spinalis
iliocostalis
X. werneri
longissimus dorsi
semispinalis-spinalis
iliocostalis
dorsal
posterior
anterior
ventral
longissimus dorsi
semispinalis-spinalis
iliocostalis
