# Supplementary material for: Evolutionary convergence of muscle architecture in relation to locomotor ecology in snakes
Source: J Anat. 2023 Feb 2;242(5):862–71. doi: 10.1111/joa.13823 (PMC10093152; doi:10.1111/joa.13823)
Supplement: Supplementary file 1 — Appendix S1 [file JOA-242-862-s001.zip › JOA_13823_Mathou et al supplementary tables.docx]

**Supplementary table 3**: principal component loadings for the analysis on non-size corrected data.

| Muscle and body dimensions | | |
| --- | --- | --- |
|  | PC1 (81.4%) | PC2 (14.3%) |
| Snout-vent length (cm) | **0.92** | -0.34 |
| Body mass (g) | **0.93** | 0.23 |
| Diameter (mm) | **0.80** | 0.51 |
| SSP length (cm) | **0.89** | -0.44 |
| LD length (cm) | **0.90** | -0.41 |
| IC length (cm) | **0.89** | -0.42 |
| SSP mass (g) | **0.94** | 0.23 |
| LD mass (g) | **0.92** | 0.31 |
| IC mass (g) | **0.88** | 0.36 |

Variables loading highly (> 0.7) are indicated in bold. IC = iliocostalis; LD = latissimus dorsi; PCSA = physiological cross-sectional area; SSP = semispinalis-spinalis.

**Supplementary table 4**: results of the MANCOVA analyses

|  | **Test** | **Value** | **F** | **Df1** | **Df2** | ***P*** |
| --- | --- | --- | --- | --- | --- | --- |
| Anterior body | | | | | | |
| Ecology | Pillai’s trace | 1.00 | 1.44 | 18 | 26 | 0.19 |
| SVL | Pillai’s trace | 0.86 | 8.42 | 9 | 12 | **0.0005** |
| Mid-body | | | | | | |
| Ecology | Pillai’s trace | 1.25 | 2.42 | 18 | 26 | **0.019** |
| SVL | Pillai’s trace | 0.72 | 3.55 | 9 | 12 | **0.022** |
| Posterior body | | | | | | |
| Ecology | Pillai’s trace | 1.01 | 1.48 | 18 | 26 | 0.18 |
| SVL | Pillai’s trace | 0.83 | 6.95 | 9 | 12 | **0.001** |

Bold values indicate significant results.

**Supplementary table 5**: results of the univariate ANCOVAs for the different body regions testing for differences between ecological groups.

| **Dependent Variable** | **Sum of squares** | **Df** | **Mean Square** | ***F*** | ***P*** |
| --- | --- | --- | --- | --- | --- |
| **Mid-body** | | | | | |
| SSP mass (g) | 0.45 | 2 | 0.23 | 1.79 | 0.19 |
| **LD mass (g)** | 1.39 | 2 | 0.70 | 4.15 | **0.031** |
| IC mass (g) | 0.74 | 2 | 0.37 | 2.66 | 0.094 |
| **SSP fiber length (cm)** | 0.42 | 2 | 0.21 | 4.29 | **0.028** |
| LD fiber length (cm) | 0.11 | 2 | 0.056 | 0.74 | 0.49 |
| IC fiber length (cm) | 0.09 | 2 | 0.045 | 0.52 | 0.60 |
| **SSP PCSA (cm^2^)** | 1.76 | 2 | 0.88 | 6.39 | **0.007** |
| LD PCSA (cm^2^) | 0.73 | 2 | 0.36 | 1.85 | 0.18 |
| IC PCSA (cm^2^) | 0.98 | 2 | 0.49 | 2.95 | 0.075 |

IC = iliocostalis; LD = latissimus dorsi; PCSA = physiological cross-sectional area; SSP = semispinalis-spinalis. Bold values indicate significant results.

**Supplementary table 6**: results of post-hoc analysis with TukeyHSD performed on the residuals of a regression of each variable against snout-vent length.

| **SSP fiber length (cm) mid-Body** | | | | |
| --- | --- | --- | --- | --- |
| **Ecology** | **diff** | **lower** | **upper** | ***P*** |
| Arboreal-Aquatic | 0.229 | -0.084 | 0.542 | 0.180 |
| Terrestrial-Aquatic | 0.180 | -0.075 | 0.435 | 0.201 |
| Terrestrial-Arboreal | -0.048 | -0.345 | 0.247 | 0.909 |
| **SSP PCSA (cm²) mid-Body** | | | | |
| Arboreal-Aquatic | -0.811 | -1.343 | -0.278 | **0.002** |
| Terrestrial-Aquatic | -0.591 | -1.025 | -0.157 | **0.006** |
| Terrestrial-Arboreal | 0.220 | -0.283 | 0.723 | 0.523 |
| **LD mass (g) mid-Body** | | | | |
| Arboreal-Aquatic | -0.906 | -1.493 | -0.320 | **0.002** |
| Terrestrial-Aquatic | -0.416 | -0.894 | 0.061 | 0.094 |
| Terrestrial-Arboreal | 0.490 | -0.064 | 1.045 | 0.089 |

Bold values indicate significant results.

**Supplementary table 7:** mean residuals for each ecological group.

| **Variable** | **Aquatic** | **Terrestrial** | **Arboreal** |
| --- | --- | --- | --- |
| SSP mass (g) anterior body | 0.33 | -0.03 | -0.47 |
| LD mass (g) anterior body | 0.49 | -0.05 | -0.66 |
| LD PCSA (cm²) anterior body | 0.47 | -0.09 | -0.57 |
| SSP mass (g) mid-body | 0.31 | -0.10 | -0.27 |
| LD mass (g) mid-body | 0.38 | -0.04 | -0.53 |
| LD mass (g) posterior body | 0.38 | -0.05 | -0.51 |
| LD PCSA (cm²) posterior body | 0.39 | -0.07 | -0.47 |

**Supplementary table 8**: results of post-hoc analysis with TukeyHSD performed on the residuals of a phylogenetic regression (PGLS) of each variable on snout-vent length.

| **Ecology** | | **Diff** | **lower** | **upper** | ***P*** |
| --- | --- | --- | --- | --- | --- |
| **SSP mass (g) anterior body** | | | | | |
| Arboreal-Aquatic | -0.809 | | -1.246 | -0.372 | **0.0003** |
| Terrestrial-Aquatic | -0.361 | | -0.717 | -0.005 | **0.046** |
| Terrestrial-Arboreal | 0.447 | | 0.034 | 0.860 | **0.032** |
| **LD mass (g) anterior body** | | | | | |
| Arboreal-Aquatic | -1.145 | | -1.708 | -0.583 | **0.0001** |
| Terrestrial-Aquatic | -0.540 | | -0.998 | -0.081 | **0.019** |
| Terrestrial-Arboreal | 0.605 | | 0.073 | 1.137 | **0.023** |
| **LD PCSA (cm²) anterior body** | | | | | |
| Arboreal-Aquatic | -1.039 | | -1.628 | -0.449 | **0.0006** |
| Terrestrial-Aquatic | -0.559 | | -1.039 | -0.079 | **0.020** |
| Terrestrial-Arboreal | 0.479 | | -0.077 | 1.037 | 0.100 |
| **SSP mass (g) mid-body** | | | | | |
| Arboreal-Aquatic | -0.580 | | -1.086 | -0.073 | **0.022** |
| Terrestrial-Aquatic | -0.412 | | -0.825 | 3.150e-05 | **0.050** |
| Terrestrial-Arboreal | 0.167 | | -0.311 | 0.646 | 0.657 |
| **LD mass (g) mid-body** | | | | | |
| Arboreal-Aquatic | -0.906 | | -1.493 | -0.320 | **0.002** |
| Terrestrial-Aquatic | -0.416 | | -0.894 | 0.061 | 0.094 |
| Terrestrial-Arboreal | 0.490 | | -0.064 | 1.045 | 0.089 |
| **LD mass (g) posterior body** | | | | | |
| Arboreal-Aquatic | -0.891 | | -1.399 | -0.382 | **0.0006** |
| Terrestrial-Aquatic | -0.430 | | -0.844 | -0.015 | **0.040** |
| Terrestrial-Arboreal | 0.460 | | -0.019 | 0.941 | 0.061 |
| **LD PCSA (cm²) posterior body** | | | | | |
| Arboreal-Aquatic | -0.862 | | -1.325 | -0.399 | **0.0003** |
| Terrestrial-Aquatic | -0.456 | | -0.833 | -0.078 | **0.016** |
| Terrestrial-Arboreal | 0.405 | | -0.032 | 0.843 | 0.072 |

Bold values indicate significant results.
