# Supplementary material for: Evolutionary convergence of muscle architecture in relation to locomotor ecology in snakes
Source: J Anat. 2023 Feb 2;242(5):862–71. doi: 10.1111/joa.13823 (PMC10093152; doi:10.1111/joa.13823)
Supplement: Supplementary file 1 — Appendix S1 [file JOA-242-862-s001.zip › JOA_13823_Supplementary captions.docx]

Figure S1: diversity of axial musculature in aquatic snakes. Illustrated are schematic representations of the iliocostalis, semispinalis-spinalis, and longissimus dorsi muscles indicated in different gray scales. vertebrae are numbered from and anterior is to the right.

Figure S2: diversity of axial musculature in aboreal snakes. Illustrated are schematic representations of the iliocostalis, semispinalis-spinalis, and longissimus dorsi muscles indicated in different gray scales. vertebrae are numbered from and anterior is to the right.

Figure S3: diversity of axial musculature in terrestrial snakes. Illustrated are schematic representations of the iliocostalis, semispinalis-spinalis, and longissimus dorsi muscles indicated in different gray scales. vertebrae are numbered from and anterior is to the right.

I also noted I forgot to update the legend of figure 4. Here is the correct one:

**Figure 4**: results of the principal component analysis performed for the anterior (A), mid-body (B) and posterior part of the body (C). The phylogeny is plotted in the morphospace. Colored symbols illustrate different ecologies. black, aquatic; white, terrestrial; grey, arboreal.
